# Effect of Mutations on mRNA and Globin Stability: The Cases of Hb Bernalda/Groene Hart and Hb Southern Italy

**DOI:** 10.3390/genes11080870

**Published:** 2020-07-31

**Authors:** Giovanna Cardiero, Gennaro Musollino, Maria Grazia Friscia, Rosario Testa, Lucrezia Virruso, Caterina Di Girgenti, Mercedes Caldora, Rosario Colella Bisogno, Carlo Gaudiano, Giuseppe Manco, Giuseppina Lacerra

**Affiliations:** 1 Institute of Genetics and Biophysics “Adriano Buzzati Traverso”, (IGB-ABT, CNR), National Research Council, 80131 Naples, Italy; giovanna.cardiero@gmail.com (G.C.); gennaromusollino@gmail.com (G.M.); 2 Azienda Ospedaliera Ospedali Civili Riuniti, Centro Trasfusionale e di Microcitemia, 92019 Sciacca, Italy; mfriscia77@yahoo.it; 3 Azienda Ospedaliero-Universitaria “Policlinico-Vittorio Emanuele”, Servizio di Talassemia ed Emoglobinopatie, 95123 Catania, Italy; testaro@libero.it; 4 ARNAS P.O. Civico e Di Cristina Benfratelli, U.O.s.d. Lab. Spec. Genetica Molecolare, 90127 Palermo, Italy; lucrezia.virruso@arnascivico.it (L.V.); caterina.digirgenti@arnascivico.it (C.D.G.); 5 P.O. Pellegrini A.S.L. Napoli1centro, 80135 Napoli, Italy; emoglobinopatie@libero.it; 6 Azienda Ospedaliera Universitaria OO. RR. San Giovanni di Dio e Ruggi D’Aragona, Medicina Trasfusionale, 84131 Salerno, Italy; rosariocolella@tiscali.it; 7 P.O. Madonna delle Grazie, Centro per la Lotta Contro le Microcitemie, ASL 4, 75100 Matera, Italy; cgaudiano@tin.it; 8 Institute of Biochemistry and Cell Biology (IBBC, CNR), National Research Council, 80131 Naples, Italy; giuseppe.manco@cnr.it

**Keywords:** human α-hemoglobin, α-thalassemia, unstable α-Hb variants, molecular chaperone AHSP, molecular modeling, mRNA quality control, no-go decay, UGT1A1

## Abstract

We identified two unstable variants in the third exon of α-globin genes: Hb Bernalda/Groene Hart (HBA1:c.358C>T), and Hb Caserta (HBA2:c.79G>A) in *cis* to Hb Sun Prairie (HBA2:c.391G>C), also named Hb Southern Italy. These mutations occurred in the H helix of the α-globin that is involved in heme contacting, specific recognition of α-hemoglobin-stabilizing protein (AHSP), and α_1_β_1_ interactions. The carriers showed α-thalassemia phenotype, but one also jaundice and cholelithiasis. Molecular identification of clusters of families in Southern Italy encouraged molecular characterization of mRNA, globin chain analyses, molecular modeling studies, and comparison with globin variants to understand the mechanisms causing the α-thalassemia phenotype. A normal amount of Hb Bernalda/Groene Hart mRNA were found, and molecular modeling highlighted additional H bonds with AHSP. For Hb Southern Italy, showing an unexpected α/β biosynthetic ratio typical of the β-thalassemia type, two different molecular mechanisms were shown: Reduction of the variant mRNA, likely due to the No-Go Decay for the presence of unused triplet ACG at cod 26, and protein instability due to the impairment of AHSP interaction. The UDP glucuronosyltransferase 1A (UGT1A1) genotyping was conclusive in the case of jaundice and cholelithiasis. Multiple approaches are needed to properly identify the mechanisms leading to unstable variants and the effect of a mutation.

## 1. Introduction

A growing number of unstable α-globin chain variants, in which the alteration of subunit folding renders the proteins susceptible to denaturation and proteolytic degradation, causes a deficit of α-chains similar to that characteristic of α-thalassemia mutations [1,2,3,4]. Some hemoglobins (Hbs) are destroyed so rapidly that they are undetectable in the hemolysate and for this reason are defined as hyperunstable. These Hbs usually result from mutations localized in the third exon, in regions coding for the α_1_β_1_ contacts that largely recapitulate the binding of the chaperone α-hemoglobin-stabilizing protein (AHSP) to the α-globin chain [5,6]. Mutations in this region were considered causative of disease because of interference with α/β-globin interaction, but discovery of the role of AHSP has suggested the interference of α-Hb/AHSP interaction as an alternative explanation [7,8,9].

Although it is generally agreed that clinical effects are related to an abnormal protein, it is plausible that in some cases the mutation may also interfere with the regulation of expression, producing aberrant mRNA that could be either inadequately processed or degraded by a mechanism of quality control [10,11].

The clarification of these aspects is important in order to understand the clinical impact of the mutations, especially if found with relatively high frequency [2].

We carried out an epidemiological study of the molecular basis of α-thalassemia in Southern Italy and identified with high relative frequencies two unstable variants: The Hb Bernalda (HBA1:c.358C>T), α1 cod 119 CCT→TCT or [α119(H2)Pro>Ser] “also named Hb Groene Hart” (throughout the paper named Hb Bernalda/Groene Hart), and the Hb Southern Italy showing the Hb Caserta (HBA2:c.79G>A), α2 cod26 GCG>ACG or [α26(B7)Ala>Thr], in *cis* to the Hb Sun Prairie (HBA2:c.391G>C), α2 cod 130 GCT>CCT or [α130(H13)Ala>Pro] [12,13,14]. These mutations occurred in the H helix of the α-globin involved in heme contact, in specific recognition of AHSP and in α_1_β_1_ interactions [7,8,9].

The Hb Bernalda/Groene Hart is the first variant with a point mutation for which a defective interaction with AHSP has been proven by in vitro experiments [15,16]. 

The first mutation in the Hb Southern Italy, at cod 26, falls close to a cryptic splicing site at codon 25–27 (ATG↓ GTGCGG>ATG↓ GTACGG), and it might cause the activation of the cryptic splicing site causing a reduction of the normal mRNA [17]. The second mutation in the Hb Southern Italy, at cod 130, studied as Hb Sun Prairie in Asian Indians, imparts Hb instability, due to the introduction of a proline residue that damages the H helix. As a consequence, the homozygous probands have chronic hemolysis [18]. 

In this study, mRNA, biochemical analysis, and molecular modeling have been performed to clarify some still open features of the mutants: We aimed to assess if the variants under study can also be Exon Splicing-affecting mutants and to elucidate the mechanisms causing the instability of the variants. The paper also shows the epidemiological distribution of the two unstable variants.

## 2. Materials and Methods

### 2.1. Families

We studied the molecular basis of the α-thalassemia in 996 families from Southern Italy. Here, we report the results of the study of 49 carriers belonging to 27 unrelated families living in Southern Italy regions (Basilicata, Sicilia, Campania, Puglia). Probands and their families were selected by the thalassemia centers collaborating in this study. A special committee of the Italian Ministry of University and Research approved the study (Decreto n. 250 of 22 June 1999) and two scientists were appointed as supervisors. The participants signed informed consents for the use of blood samples. Ethical approval of the protocol was obtained from the Comitato Etico Università Federico II (307/2016).

### 2.2. Hematological Data

Hematological parameters, ferritin, transferrin, serum iron, zinc protoporphyrin (Zpp), and bilirubin (total and indirect) were determined by standard methods in the collaborating hospitals. The analysis of hemoglobin (Hb) was performed by cation exchange high performance liquid chromatography (HPLC) (Bio-Rad, Diamat or Variant System Hercules, CA, USA). Heinz body formation and thermal and isopropanol Hb stability tests were performed by standard methods.

### 2.3. Globin Chain In Vitro Biosynthesis

In vitro biosynthesis of globin chain in reticulocytes was performed in a carrier of Hb Southern Italy and in a normal control, as already reported. Globin chains were separated by reversed-phase HPLC [19].

### 2.4. DNA Analysis

Molecular analysis for the α-thalassemia mutations were performed according to previously reported protocols [13,19,20]. Proper amplification-refractory mutation system (ARMS-PCR) assays for the definition of the heterozygous Hb Bernalda, Hb Caserta, and Hb Sun Prairie genotypes were set up [13]. The analysis of the three SNPs, RsaI 5’ of the α2-globin gene (rs2541669), α2 + 14 (HBA2:c.−24C>G rs772829778), and α2 + 861 at the 3’ UTR of HBA2 (HBA2:c.* 136A>G, rs2685121) were performed as previously reported [19,20,21,22]. The analysis of the polymorphism in the promoter of the UGT1A1 gene was carried out by sequencing. All oligonucleotides are reported in Table S1 [23,24].

### 2.5. mRNA Analysis

Purification of mRNAs, from reticulocyte-enriched peripheral blood cells and from peripheral blood stem cells (PBSC) were differentiated in vitro, as well as reverse transcriptions were performed according to references [21,22]. The qualitative and semi-quantitative cDNA analyses, specifically sequencing, separation on acrylamide gel, BstNI restriction enzyme digestion, and double-gradient denaturing gradient gel electrophoresis (DG-DGGE) separation, were performed as already reported [21,22].

### 2.6. PBSC Differentiated In Vitro

The separation and differentiation in vitro of the PBSCs, from one normal subject and one carrier of Hb Southern Italy, and the separation and extraction of the RNA from the nuclei and cytoplasm were performed as published elsewhere [21,22].

### 2.7. Database

All data regarding the families under study and the experimental results were collected in an anonymous form in a database developed on the Microsoft Visual Fox 6.0 platform and interfaced with an external software such as Microsoft Excel and Microsoft Word (Microsoft, Redmond, WA, USA) [25].

### 2.8. Software and Structural Analysis

Secondary structure predictions of the mutant α-chains were obtained by means of the SOPMA web application (https://npsa-prabi.ibcp.fr/cgi-bin/npsa_automat.pl?page=npsa_sopma.html). The software Splice Site Prediction by Neural Network (https://www.fruitfly.org/seq_tools/splice.html) was used for the prediction of splicing sites. The secondary structure of the mRNA was predicted by means of the RNAfold web server (http://rna.tbi.univie.ac.at/cgi-bin/RNAWebSuite/RNAfold.cgi) [26]. We evaluated mutation-induced structural alterations by analyzing the structure of α-chain of human hemoglobin in the complex with AHSP (PDB code 1Y01) and in the tetrameric α_2_β_2_ structure (PDB code 2HHB) [3,27]. Structural analyses were performed by using the programs Yasara with FoldX 4.4.23 (http://foldxsuite.crg.eu/, CRG, Barcelona, Spain) add-on and Swiss PDB viewer (www.expasy.org) [28]. Known data on the codon usage were obtained by using the software (https://www.kazusa.or.jp/codon/cgi-489 bin/showcodon.cgi?species=9606) [29].

## 3. Results

### 3.1. Hb Bernalda

**Figure 1:**
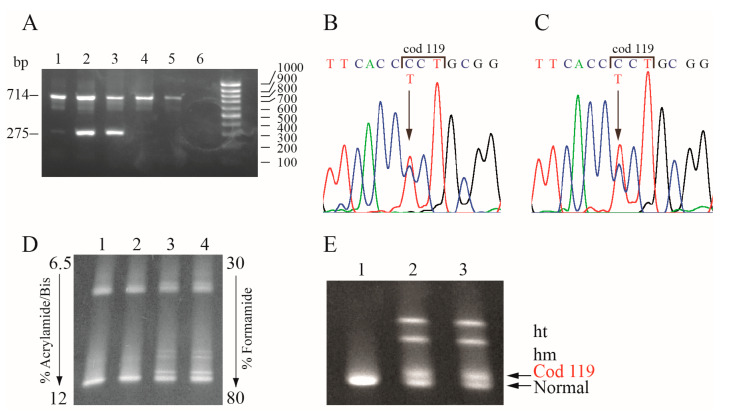
Molecular characterization and cDNA analysis of the Hb Bernalda. (**A**) ARMS for the molecular identification of the α1 cod119 CCT>TCT mutation (Hb Bernalda); the control amplicon was of 714 bp, the mutation specific amplicon was of 275 bp. Hb Bernalda ARMS positive samples have to show a 275 bp band with a threshold intensity of 15% relative to the 714 bp control band. Lanes 1, 4, and 5: Normal controls; lanes 2 and 3: Hb Bernalda heterozygotes; Lane 6: Negative control, no DNA. (**B**) and (**C**) α1 gDNA and cDNA sequences, respectively, of a carrier of the Hb Bernalda from codon 117 to codon 121. The arrow indicates the mutation. (**D**) and (**E**) DG-DGGE of the gDNA and cDNA fragment containing exon III, respectively, of the α-globin gene. (**D**) Lanes 1 and 2: Normal subjects; lanes 3 and 4: Hb Bernalda heterozygotes (**E**) Lanes 1: Normal subject; lane 2 and 3: Hb Bernalda heterozygotes. The semi-quantitative analysis was performed on cDNA from Hb Bernalda carriers as follows: Amount of Hb Bernalda cDNA = Mutant Homoduplex + (sum of the two heteroduplex bands/2); amount of normal cDNA = Normal Homoduplex + (sum of the two heteroduplex bands/2).

The Hb Bernalda was identified in 17 unrelated families from Southern Italy in a total of 29 carriers and 1 compound heterozygote for the α-thalassemia α1 cod23 GAG>TAG (HBA1:c.70G>T). Six families were originating from Basilicata, in particular one from Potenza and five from two villages located in a restricted area between Matera and Jonian coast of Metaponto. The variant was called Hb Bernalda because it was identified for the first time in a carrier from this village [12,15]. The 11 families from Sicily come as nine from Sciacca, one from Catania, and one from Palermo. The 24 carriers had a mild reduction of the MCV (range 69.8–82.8, average 77.5) and MCH (range 22.8–27.4, average 26.0) associated with a normal Hb A2 (Table 1). Two heterozygotes over 70 years old, two infants, one sideropenic patient, and the compound heterozygote, were excluded from the hematological analysis. HPLC, electrophoretic separation, and spectrometric mass analyses did not show the presence of anomalous hemoglobins. The isopropanol and thermal stability test did not show any precipitation. In all the 17 families, the mutation was highlighted by DG-DGGE (Figure 1D) and confirmed by sequencing and ARMS protocols (Figure 1A,B).

A molecular modeling approach was used to try to understand the structural effect of the Pro119Ser mutation in the protein structures 1Y01 and 2HHB (Figure S1A,B, respectively).

**Figure 2:**
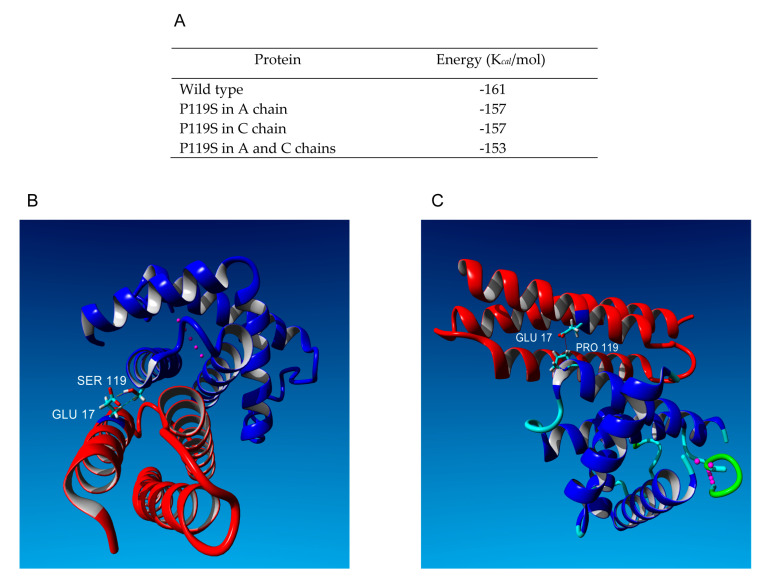
(**A**) FoldX stability analysis of the tetrameric hemoglobin with the α-chain variant Hb Bernalda/Groene Hart in comparison with the wild-type (2HHB). (**B**) Model of the Hb Bernalda/Groene Hart mutation in 1Y01 showing ionic interactions with GLU17. (**C**) Wild-type 1Y01 showing the PRO119 hydrophobic interaction with GLU17.

The wild-type structure was first “repaired” with the specific FoldX tool. The heme moiety, water, and other heteroatoms were removed. Then, we inserted the mutation in one or two α-globin chains of the tetramer (pdb: 2HHB) and calculated the stability by the FoldX algorithm running under the Yasara program. The root mean square deviation (RMSD) between the wild-type and the oligomer with the two mutated α-globin chains was 0.009 Å on 4970 matched atoms meaning a very limited global effect. FoldX was used to calculate the stability of the repaired wild-type and the three minimized mutants. The values obtained were reported in Figure 2A. Clearly the mutation has a destabilizing effect and in an additive way.

Then, we moved to analyze the effect of the mutation in the interaction with AHSP. 

Again, the wild-type complex (pdb: 1Y01) was repaired by FoldX and the new mutant created. The RMSD between the two objects was 0.14 Å over 3355 matched atoms thus indicating a higher conformational change due to the mutation with respect to the Hb oligomer. By the FoldX analysis the overall stability of repaired wild-type was 71.04 kcal/mol whereas that of the mutant was 74.18 kcal/mol. In the mutant, the interaction energy at the interface between the A and B chain was −16.44 kcal/mol against −18.79 of the wild-type, again indicating global destabilization. A careful analysis of the structures with the Yasara program allowed seeing significant differences in terms of broken bonds or interactions. We observed that a hydrophobic interaction between Pro119 and Tyr51 in the wild-type disappeared and was substituted by ionic interactions of Ser119 with Glu17 of the mutant. The Swiss PDB viewer program also was used to highlight hydrogen bonds (Figure S2A). In this case, additional differences were observed in the region of the mutation. The Thr118 before the mutated proline formed a weak hydrogen bond (3.2 Å) with AHSP Tyr51 (missing in the wild-type; Figure S2B) and a rotamer of Ser119 formed a hydrogen bond with AHSP Tyr48 (Figure S2C). These interactions were absent in the mutated 2HHB. The only differences that we detected in this model concerned H bonds at the subunits’ interface involving Arg130 and His116 of the β-chain that appeared destabilized in the sense that bonds in which residues were involved were mutually exclusive depending on rotamers. This is in line with the stability measurements (Figure 2A).

We concluded that new interactions between AHSP and the mutated α-globin chain overall destabilize the complex by disrupting multiple interactions elsewhere and the same seems to happen in the Hb oligomer. 

To assess if the Hb Bernalda could be splicing-affecting genomic variants (SpaGVs), inducing exon skipping or activation of cryptic splice sites, we performed a qualitative and quantitative mRNA analysis [10]. The analysis of Hb Bernalda with software for the prediction of alternative splicing sites gave negative results. This prediction was confirmed by the separation on acrylamide gel of full-length α-globin cDNA (data not showed) and of an ExII-ExIII cDNA fragment amplified using ^32^P dCTP revealing the absence of anomalous bands and aberrant splicing. The sequencing of the α1-globin gene cDNA from reticulocytes revealed the presence of mutated cDNA (Figure 1C). The anomalous peak T was of similar intensity to the normal C. To assess if the mutant mRNA underwent a quality control system triggering a degradation pathway and to evaluate the level of mutant cDNA, we performed the semi-quantitative analysis of the homoduplex and heteroduplex cDNA bands following DG-DGGE separation [21,22]. As shown in Figure 1E, the four bands had similar intensity. Analysis of the data by the KodaK Carestream MI software indicated that the amount of Hb Bernalda mRNA in the reticulocytes from peripheral blood was comparable to the normal α2-globin mRNA (Figure 1E) [21]. 

The analysis of the three non-pathogenic polymorphisms rs2541669, rs772829778, and rs2685121, very close to or inside the α-globin genes, carried out as reported in Section 2.4, showed the same haplotypes “+ − −” in all the 17 families.

#### Family 1

The proband of family 1 was compound heterozygous for two rare mutations localized in the α1-globin gene: The Hb Bernalda and the non-sense mutation α1 cod23 GAG>TAG. The proband and the mother showed a higher level of Hb A2 (3.2%), but the restriction mapping analysis excluded the presence of α-globin gene triplication, and the sequencing of the β-globin gene of the mother did not reveal the presence of point mutations [22]. Both the defects cause a decrease of α-chain synthesis, leading to the α-thalassemia phenotype (Table 1).

### 3.2. Hb Southern Italy

The gene showing association in *cis* between the Hb Caserta and the Hb Sun Prairie was identified in 10 unrelated families in a total of 18 carriers. Seven families were originating from Campania, two from Sicily, and one family from Basilicata, all regions of Southern Italy. In all the families, the heterozygotes showed mild microcythemia (MCV 74.6 ± 2.2, range 71.0–78.6; MCH 24.9 ± 0.7, range 23.6–26) with normal iron metabolism, Hb A2 in the normal range, and no observation of Hb variants by cation-exchange HPLC or cellulose acetate electrophoresis (Table 2).

**Figure 3:**
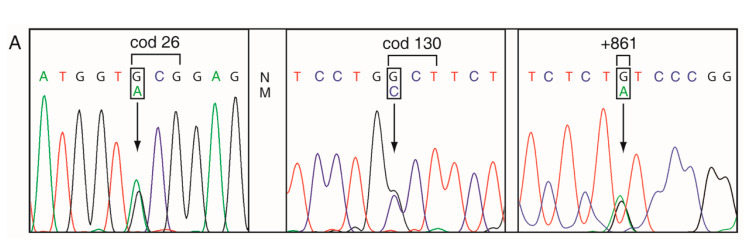

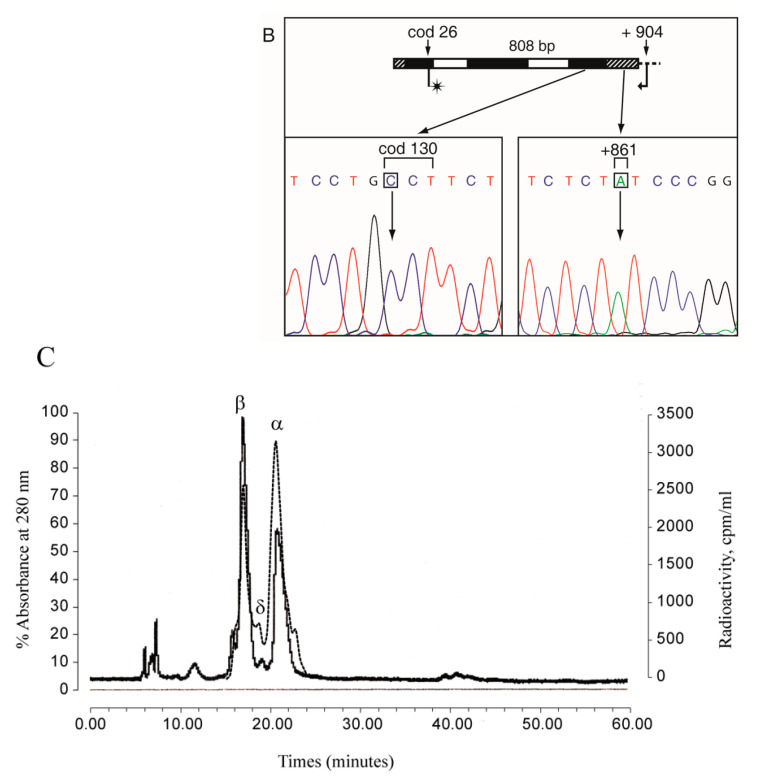
Phasing of the allele Hb Caserta in *cis* to the Hb Sun Prairie. (**A**) The sequencing of the α2-globin gene (genomic DNA) of a carrier of the Hb Sun Prairie showed that it was heterozygous also for the Hb Caserta and for the SNP α2 + 861 G>A, but not for the α2 + 36 C>T [30]. (**B**) The sequencing of the mutant ARMS-amplicon amplified from the same carrier with the ARMS-primer specific for the mutation Hb Caserta and spanning the allele up to the 3’ UTR region showed that the two mutations and the SNP were associated in *cis.* (**C**) Globin chain separation by reversed-phase HPLC in the blood of an Hb Southern Italy heterozygote. The α-, δ-, and β-globin chains are reported. The broken line indicates the counts per minute (cpm) of the globin chains.

The DG-DGGE analysis identified two anomalous patterns, respectively, in the first and third exons of the α2-globin gene as previously reported [20]; the sequence analysis showed the presence of the mutation α2 cod26 GCG > ACG and α2 cod130 GCT > CCT and the presence of the SNP + 861 G > A in the carriers (Figure 3A), while no other mutations were detected in the α1-globin genes. In addition to the carriers, fifteen heterozygotes and three compound heterozygotes, two for the −α^3.7^ deletion (NG_000006.1:g.34164_37967del3804) and one for the α2^IVSI-5nt^ (HBA2:c.95+2_95+6delTGAGG), were identified.

To investigate the molecular mechanism that underlies the α-thalassemia phenotype associated with Hb Southern Italy, we examined the variant both at the protein and mRNA level. In particular we performed: (a) The biosynthesis in vitro of the globin chains; (b) a meta-analysis using software for the prediction of alternative splicing; (c) sequencing and qualitative analysis of the α-globin mRNA both from the reticulocytes and from PBSC differentiated in vitro to identify the presence of alternatively spliced mRNA; (d) semi-quantitative analysis by digestion with the restriction enzyme BstNI to quantify the amount of the mutant cDNA at codon 130 GCT>CCT.

The globin chain biosynthesis in vitro was carried out with a 120 min incubation for the carrier n. 18 (Table 2). No abnormal peak of OD at 280 nm or of ^3^H-cpm was revealed by HPLC (Figure 3C). The non-α/α ratio of OD at 280 nm was in the normal range. The ^3^H-specific activity (^3^H-cpm/mg) of α-globin chains (37,672) was higher than that of the β-chains (30,756) and the α/β ratio was 1.39 (1.23 and 1.56) and therefore typical of a mild β-thalassemic type (Figure 3C). 

**Figure 4:**
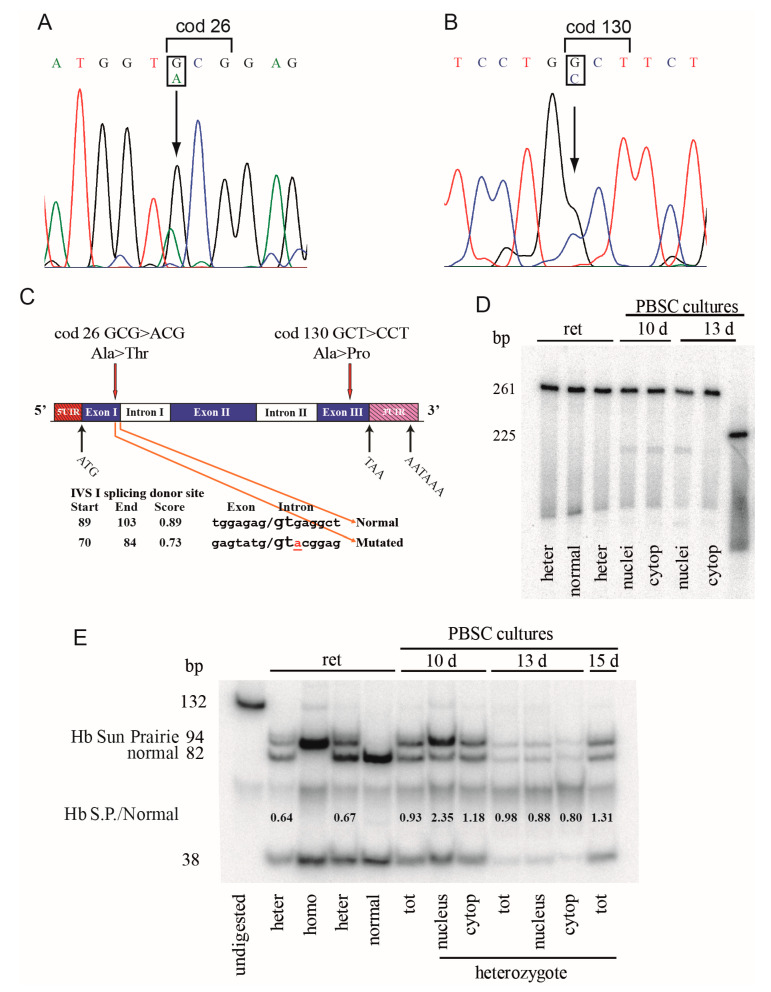
Hb Southern Italy cDNA analysis. (**A**,**B**) The α2 cDNA sequence of a carrier of the Hb Southern Italy showed the presence of both the mutations at codon 26 and at codon 130. The arrows indicate the mutations. (**C**) Scheme of the α-globin gene and of possible splicing. The prediction of splicing, the positions, the sequences involved, and the scores are reported. (**D**) Electrophoresis on denaturing acrylamide gel of ^32^P cDNA fragments containing the exon I of the α2-globin gene from Hb Southern Italy heterozygotes and normal subjects. ret: Reticulocytes; 10 d and 13 d indicate the days of the erythroid cultures. (**E**) Digestion of the exon III cDNA of the α2-globin gene with the restriction enzyme BstNI that recognizes the Hb Sun Prairie (Hb S.P.) mutation. The α2 cDNA BstNI digestion displayed two bands in normal subjects (82 and 38 bp), and one additional undigested band of 94 bp (82 + 12), in the Southern Italy carriers, while the expected 12 bp band was too short to be detected by electrophoresis. N: Nucleus; C: Cytoplasm; tot: Total; 10 d, 13 d, and 15 d indicate the days of the erythroid cultures; ret: Reticulocytes from carriers, homozygotes, and normal subjects. The ratio Hb Southern Italy(94 bp)/Normal(82 bp) is reported in the lower section.

Sequencing of the cDNA of carriers of Hb Caserta in *cis* to Hb Sun Prairie showed heterozygosity at cod 26 (G>A) and at cod 130 (G>C) (Figure 4A,B); the anomalous peaks A and C were smaller than the normal G, suggesting a reduction of the mutant mRNA; this assumption was investigated by different approaches. The software analysis for the prediction of splicing sites revealed that the mutation at cod 26 creates a splicing donor site with a score-splicing consensus (0.73) lower than the normal site (0.89) (Figure 4C). The separation of the cDNA fragment on a polyacrylamide gel showed two cDNA fragments, the first of normal length (261 bp) and the second of about 210 bp, in the nuclei and cytoplasm of BFU/CFU at 10 and 13 days of differentiation and in the reticulocytes (Figure 4D). The shorter band of 212 bp had the length generated from the constitutive alternative splicing at cod 15, as previously demonstrated [22]. The anomalous fragment of 242 bp, expected in the case of splicing at codon 25–27, was not observed also at higher time of exposure. 

To check the rate of synthesis and the stability of the cDNA with the two mutations in *cis*, the ratio of mutant/normal cDNA was established by means of an analysis with the BstNI restriction enzyme, for which the mutation at cod130 eliminates the site 5’-CC^WGG-3’. The ratio codon 130/normal mRNA was close to 1 in the total sample from the PBSC cultures at 10, 13, and 15 days (0.93, 0.98, and 1.31), and 0.64 or 0.67 in the reticulocytes of the two carriers, respectively (Figure 4E). The result from the nuclei at 10 days was not informative due to the excess of undigested samples, and the data from cytoplasm at 10 and 13 days of differentiation were near to 1 (1.18 and 0.8). These data suggest that: a) In the nucleus, the variant mRNA is synthesized in an equal amount relative to the normal mRNA; b) most likely about 35% of the variant mRNA synthesized in the nucleus is subsequently degraded, leading to its reduction in the reticulocytes. 

The instability of the Hb Southern Italy (α2 Ala26Thr in *cis* to Ala130Pro) indicated by the absence of the variant hemoglobin and of the variant α-chain, was investigated by biochemical and molecular modeling approaches. 

In the structure of normal human hemoglobin, the Ala26 on the B Helix (B2) is an internal partly exposed amino acid whereas the Ala130 on the H helix (H13) is close to the central cavity, taking contacts inside the H Helix and likely not affecting the α−β-interface interactions (Figure S1D). The position of the two residues in 1Y01 is also shown in Figure S1C. 

The structural analysis by molecular modeling approaches in 2HHB (Figure S1D) revealed the absence of variation in the interaction between the B and H helix, and a slight variation of stability in the formation of the Hb tetramer with a destabilization of 6 kcal/mol (Table 3). The data are partly in agreement with those reported in previous molecular modeling applied to the Hb Sun Prairie, indicating no severe structural distortion in the Hb molecule because of the substitution [30]. 

The residues 26 and 130 of the α-globin chain are reported not to be involved in the direct interaction with AHSP [5,6].

The molecular modeling prediction of the interaction shows that in the 2HHB oligomer there is a slight decrease of stability that has to be ascribed entirely to chains A and C (Table 3). In the C chain of the oligomer three residues move substantially in terms of RMSDs with respect to the wt, namely: Lys60 (0.411 Å), Lys127 (0.1745 Å), Leu129 (0.2275 Å). In A only Lys127 (0.165 Å) moves substantially. It is worth noting that all those residues but Lys60 are involved in the interface between the A and C chains (ValA1, AspA6, AlaA123, AspA126, LysA127, ProA130, ArgA141, ValC1, AspC6, AspC126, LysC127, ProC130, ArgC141) and comprise the mutation at position 130.

The analysis of the two non-pathogenic polymorphism α2 + 14 and α2 + 861 underlined the same haplotype “− +” in all the 10 families indicating that very likely the origin of Hb Southern Italy is unique (Table 2).

#### 3.2.1. Family 19

Family 19 was sent to our observation for the presence of microcythemia in the proband (MCV 65.2, MCH 21.3), brother (MCV 73.9, MCH 25.0), and father (MCV 78.6, MCH 26.0) associated with the normal level of iron and Hb A2. The proband showed visible jaundice in the skin and mucous membranes. The first major episode of jaundice had occurred at the age of 18 during the military service. The external observation indicated an increased size of liver and spleen that protruded 1.5 and 4 cm, respectively, from the costal arch. The ultrasound examination showed normal liver, normal bile ducts, but cholelithiasis and an enlarged spleen. The direct and indirect Coombs test was negative, the test on the G6PD enzyme activity was normal and the Heinz bodies were absent. The analysis of the deletional mutations was positive for mother and proband, who were heterozygotes for the −α^3.7^ deletion. The sequencing analysis of the α2-globin gene showed the presence of the Hb Southern Italy in hemizygosis in the proband and in heterozygosis in the father and in the brother. The α1-globin gene sequence was normal. 

**Figure 5:**
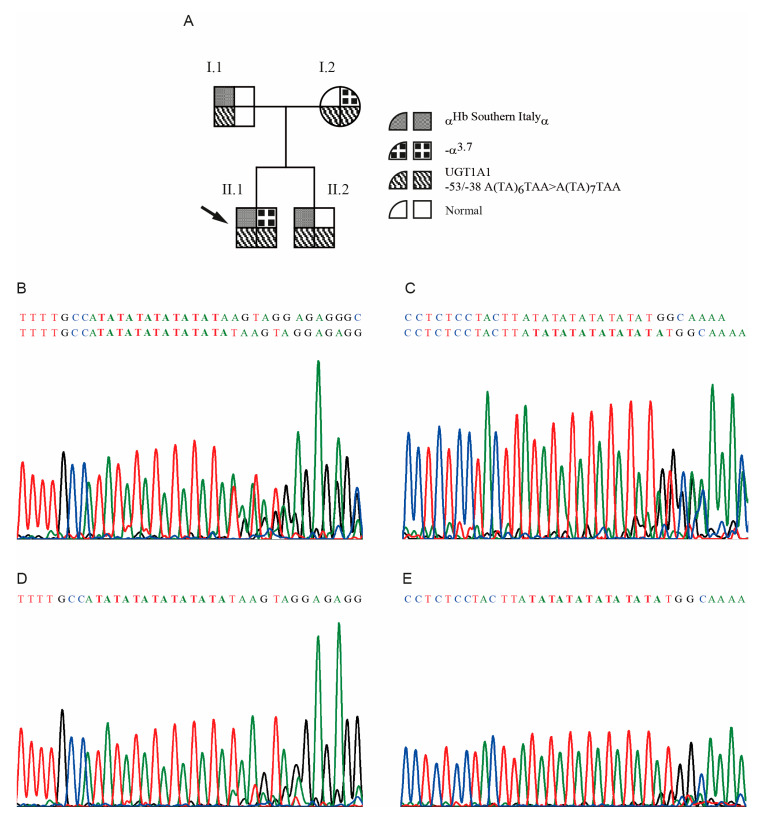
Family 19. (**A**) Pedigree of family 19. The arrow indicates the proband. The genotypes of the α-globin genes and of the *UGT1A1* promoter, for each member of the family, are reported. (**B**) and (**C**) Sense and anti-sense DNA sequences, of the subject I.1 of family 19, respectively. The frameshift indicates that the subject was heterozygote for the alleles A(TA)_6_TAA/A(TA)_7_TAA. (**D**) and (**E**) Sense and anti-sense DNA sequences, of the patient II.2 of family 19, respectively, showing the homozygosis for the allele A(TA)_7_TAA/A(TA)_7_TAA.

To investigate the considerable increase in bilirubin in the proband (tot 5.3, ind 4.9), which was modest in the mother (tot 1.7, ind not available) and brother (tot 1.1, ind 0.9), we carried out the analysis of the *UGT1A1* gene, to evaluate the presence of mutations causing Gilbert’s Syndrome [23,24]. The sequence analysis of the *UGT1A1* promoter showed the presence of the allele A(TA)_7_TAA in homozygosis in the proband, the mother and the brother (Figure 5D,E), while the father was heterozygote A(TA)_6_TAA/A(TA)_7_TAA, as shown in Figure 5. 

#### 3.2.2. Family 23

Family 23 was sent to our observation for the presence of severe and mild microcytosis, in the proband and in the parents, respectively. The phenotype was associated with a normal level of Hb A2 and iron. The molecular characterization showed that the proband was compound heterozygous for the point-mutational α-thalassemia α2^IVS1-5nt^, inherited from the mother, and the Hb Southern Italy from the father. The proband showed anemia (Hb 10.5 g/dL) associated with reduction of MCV (64 fL) and MCH (20 pg), but jaundice was not reported (Table 2). 

### 3.3. Epidemiology and Origin of the Mutants

The two variants displayed a relatively high frequency in the Southern Italy regions as shown in Table 4.

The Hb Bernalda was present in 17 out of 996 families studied in our project in which 1092 chromosomes with α-thalassemia mutations were identified. These data could indicate that the relative frequency of Hb Bernalda is about 1.6% in Southern Italy. Moreover, the analysis of the patients’ region of origin pointed out the presence of two clusters of families, on the Ionic cost of Italy and on the Southern cost of Sicily, respectively. In Matera, the Hb Bernalda was identified in five families showing a relative frequency of 2.0%, which increases to 7.9% considering only the point mutations. In Sicily, the Hb Bernalda was identified in 11 families with relative frequency of 1.8% corresponding to about 6% in the case of point mutations. More interestingly, limiting the analysis to the Agrigento area, the relative frequency increases to 4.6% and up to 16.1% for point mutations.

The Hb Southern Italy showed in our sample a frequency of 0.9%. The analysis of the region origin indicated also in this case the presence of two clusters of families, in Campania and Sicily, respectively. The Hb Southern Italy showed in Campania a frequency of 3.2% increasing to 20.0% when taking into account the point mutants, only; in Sicily the double mutants showed a lower frequency of 0.5% and 1.6%, respectively. The data on the two clusters of families is confirmed by others authors, one describing the Hb Southern Italy, in three families from Campania and three from Sicily, respectively, and the second describing it in a single subject from Sicily [31,32]. Moreover, two of the patients from Sicily were homozygotes for the Hb Sun Prairie, confirming the presence of a cluster of families [31]. 

## 4. Discussion

In this study, through a multidisciplinary approach we contribute to increase our knowledge on the effects of altered interactions of variant α-globin chains with the AHSP chaperone, and on the activation of mRNA quality control mechanisms, which confirm the importance of globin genes as model systems in the study of hereditary diseases [9,10].

### 4.1. Hb Bernalda

The Hb Bernalda also known as Hb Groene Hart is a hyperunstable α-globin variant due to the substitution α1 119(H2)Pro>Ser. This variant was supposed to not be produced in erythrocytes of the carriers because it could not be identified with conventional procedures. The α119(H2)Pro forms α_1_β_1_ contacts in the human hemoglobin and is invariant in all previously sequenced human α and non-α chains, except for embryonic ζ-chains, where it is replaced by Ile [33]. This α-globin mutation has been studied in detail in order to demonstrate how point mutations may cause α-thalassemia by affecting interactions with the chaperone AHSP [7,8,16]. Co-expression of α-globin with AHSP or β-globin in *Escherichia coli* indicated that alterations at α-globin amino acid positions 103, 117, and 119 affect both β-globin and AHSP interactions, providing at least two mechanisms for their destabilizing effects [7,8].

In the present article, we performed for the first time in silico stability measurements and analyzed the interactions of Hb Bernalda with AHSP and the β-globin chain, highlighting that the mutant α-globin likely forms a weak H bond between the Thr118 and AHSP Tyr51 (3.2 Å, missing in the wild-type; Figure S2B) and one rotamer of Ser119 forms an H bond with AHSP Tyr48 (Figure S2C). These interactions were absent in the mutated 2HHB, in which the only differences that we detected were in the H bonds at the interface involving Arg130 and His116 of the β-chain, which appeared destabilized. The data were in line with the stability measurements. 

The mutant α-chains affecting the interaction with AHSP are usually characterized by a rapid degradation, and for this reason are not always identified [34]. In the case of Hb Bernalda, the formation of further H bonds, as the in silico analysis suggested, could stabilize the interaction with AHSP and explain the chain recovery as an abnormal peak, as reported by Zanella et al. [35]. 

In the present article, we show for the first time the absence of aberrant splicing at biologically relevant levels, and by semi-quantitative analysis we demonstrated that the amount of Hb Bernalda cDNA in the reticulocytes from peripheral blood was comparable to the normal α1-globin mRNA (Figure 1E) [21]. These data excluded the presence of mechanisms that could reduce the amount of the variant mRNA. Therefore, the abundant mRNA detection establishes that the pathological phenotype is at the protein level, most likely based on impaired interaction with AHSP (Figure 2B,C) [16]. 

At the clinical level, it is relevant to note that the association of a highly unstable α-globin variant with α^0^ or α^+^ thalassemia can cause a relatively severe dyserythropoietic anemia. This is not the case for the proband of family 1 (Table 1), who was compound heterozygous for the two rare mutations (αα^Hb Ber^/αα^cod23^) showing hematological alterations of α^0^ thalassemia with a normal level of hemoglobin. 

The Hb Bernalda/Groene Hart has been described in Southern Italy, Sicily and in North Africa, Morocco, Algeria, Tunisia, and recently in Spain. Lately, the Hb Macarena (HBA2:c.358C>T) has been described, showing the same substitution as Hb Bernalda, but associated with the α2-globin gene [36]. 

Due to the proximity of these territories and the frequent incursions of Berber and Saracen populations on the Italian coasts, a unique origin and subsequent spread throughout Mediterranean countries could be suggested for this mutation. 

We also demonstrated the unique origin of Hb Bernalda in Southern Italy, following the identification of the same haplotype in all families [21,22]. It could be interesting to analyze the same SNPs in African carriers to assess if the origin is unique also between the two continents. 

### 4.2. Hb Southern Italy

The Hb Southern Italy (Hb Caserta in *cis* to Hb Sun Prairie) is a rare example of the association of two mutations on the same α-globin gene, described in Italian patients in 2007 [13,14,37]. Actually, we performed several studies to clarify the processes eventually leading to the α-thal phenotype.

The biosynthesis in vitro in a carrier of the Hb Southern Italy unexpectedly showed that the α/β biosynthetic ratio was 1.39 and consequently in favor of a β-thal genotype. This value of the β-thalassemia type is frequent in unstable variants and has already been reported for the Hb Sun Prairie [18,19]. The present moderate alteration of the biosynthetic ratio relative to the Hb Sun Prairie (α/β ratio 2.1–2.3) [18]) could be due to the presence in *cis* of the Hb Caserta that could make the globin chain more unstable and thus cause rapid degradation. The separation on HPLC did not reveal any abnormal peak by OD measurement at 280 nm or by measurement of ^3^H-cpm (Figure 3), confirming the rapid degradation of Hb Southern Italy, in contrast to Hb Sun Prairie, which results in an anomalous α-globin chain at 3% to 5% of total hemoglobin [38].

Molecular modeling studies of the hemoglobin tetramer of Hb Southern Italy and of the AHSP-α-globin chain complex revealed instability of 6 kcal/mol in the Hb tetramer. In particular, destabilization in the tetramer affects exclusively the A and C monomers that harbor mutations (4 and 2 kcal/mol in A and C, respectively). By looking at the interaction of the α-globin chain with AHSP, the interaction contacts between the two polypeptides appeared identical in the wild-type and in the mutant.

To try to explain the reason of instability we looked at the mutants in the H helix of the α- and β-globin chains. In the case of Hb Utrecht (α2cod129 Leu>Pro, HBA2:c.389T>C) the instability is caused by an impaired interaction with AHSP [7]. Analysis of the effect of deletion in the H helix revealed that amino acids 129, 132, and 136 are involved in the interaction with heme [9]. Finally, out of the β-globin variants, the Hb Altdorf (β135(H13)Ala>Pro, HBB:c.406G>C), showing the same Ala>Pro substitution as the Hb Sun Prairie [39], although deficient in heme, was identified as about 11% of hemoglobin variant and 35% of abnormal globin chain. These data from H helix mutants could suggest that most likely the absence of the Hb Southern Italy is attributable to the mutation at residue 130 in a context important for both the interaction with heme and with AHSP, the absence of which could cause rapid degradation.

The analysis of mRNA was performed to assess the presence of factors that could reduce the amount of the double mutant at cod26 and at cod130. For the quantitative analysis we focused the attention on both the mutations, but utilizing different approaches. In the case of the Hb Caserta no abnormal length mRNA from the mutated sequence was detected, but only expected mRNAs spliced at cod31 and at cod15 (Figure 4D) [22]. This means that the supposed alternative splicing at cod26 showing a score of 0.73 is not active (Figure 4C) and the mutation did not affect the amount of the mutant mRNA. The semi-quantitative analysis by digestion with the restriction enzyme BstNI, that recognizes the normal sequence at cod130, carried out on reticulocytes and PBSC differentiated in vitro, showed that the Hb Southern Italy mRNA was comparable in amount to the normal mRNA at 10, 13, and 15 days of differentiation (abnormal: Normal mRNAs = 0.93, 0.98, and 1.31), the data from nucleus and cytoplasm confirming the observation on total fraction. Moreover, a reduction of about 35% of the mutant mRNA was observed in the reticulocytes from peripheral blood (Figure 4E). This reduction could be a consequence of activation of the No-Go Decay, one of the several distinct mechanisms that control the quality of mRNAs and proteins during translation at the ribosome, to reduce the toxic effect of aberrant proteins, and resulting in many human diseases [10,11]. 

It could be interesting to compare the amount of mRNA for variants described in close proximity to the cod130 to define if there is a consensus sequence involved in mRNA degradation, causing a reduction of variant globin chains. The analysis of the percentage of stable Hb variants at cod130 (Hb Yuda, HBA2:c.392C>A = 30%, Hb Westborough, HBA1:c.392C>T = 30%) or in close proximity at cod131 (Hb Lusaka, HBA1:c.395C>T = 20%, Hb Cap-d’Agde, HBA2:c.395C>G = 24%) indicated that they were present in an expected normal amount [3,4]. These data might indicate that only specific mutations are able to affect mRNA levels.

The genetic code is degenerate as most amino acids are encoded by multiple synonymous codons, but the codons are not equally utilized. Known data on codon usage in homo sapiens (https://www.kazusa.or.jp/codon/cgi-bin/showcodon.cgi?species=960) [29] indicated that codon ACG–Thr is poorly used being the fourth last codon by frequency (6.1‰), excluding the stop codons. Out of the 19 amino acids present in the α-globin chain, threonine is decoded only by ACC(9), while ACT(0), ACA(0), ACG(0) are absent (Table S2). Analyzing the three mutant codons, it is evident that cod119TCT-Ser (Hb Bernalda) and cod130CCT-Pro (Hb Sun Prairie) are decoded by triplets normally present in the α-globin mRNA; on the contrary, the cod26ACG-Thr of Hb Caserta is not present in either the α- or β-globin mRNA (Tables S2 and S3). In addition, the ACG codon can be used as a non-canonical transcriptional start site [40]. Any slowdown in the synthesis caused by the presence of the ACG codon could therefore cause the activation of the No-Go Decay [11]. The analysis of the mRNA secondary structure [26] of the double mutant relative to the normal α2-globin mRNA also highlighted several differences in the conformation that could alter the accessibility to the mRNA, as shown in Figure S3. In the case of the Hb Southern Italy, two mechanisms could activate the No-Go Decay: Introduction of a rare codon (ACG) and the alteration of the mRNA structural conformation, favoring the stalling of ribosomes during translation [11,40].

Another interesting aspect was the impact on clinical manifestation in the presence of modifier genes. Two compound heterozygotes have been identified, the probands II.1 of family 19 (α^Hb SI^α/−α^3.7^) and II.1 of family 23 (α^Hb SI^α/α^IVSI-5nt^α). The proband of family 23 showed anemia (Hb 10.5 g/dL) because the allele α^IVSI-5nt^ is more severe than the −α^3.7^ and therefore causes a higher percentage of unstable globin variant. In contrast, although the proband of family 19 had a normal level of Hb (Hb 12.8 g/dL), it showed a more severe phenotype due to the high level of bilirubin and cholelithiasis. The genotype of the UGT1A1 gene showed the presence of homozygosity for (TA)_7_, justifying the high level of bilirubin and cholelithiasis in the proband and the lowest level of bilirubin in the mother and in the brother [23,24]. In particular, the brother heterozygous for the Hb Southern Italy and homozygous for the UGT1A1 (TA)_7_ shows an acceptable level of bilirubin and absence of cholelithiasis, indicating that the co-presence of –α^3.7^ deletion increases the percentage of the unstable variant and thus the severity of the phenotype. Therefore, this study confirms that, in the case of unstable variants, *UGT1A1* genotyping is a useful tool for identifying individuals with hemoglobinopathy at high risk of cholelithiasis and requiring close clinical monitoring [41].

## 5. Conclusions

In summary, we demonstrated that the α-thalassemia identified in some clusters of families was associated with two unstable variants Hb Bernalda/Groene Hart and Hb Southern Italy, which were located in the H helix of the α-globin gene. 

Our analyses and modeling predictions for the ASHP-Hb Bernalda/Groene Hart interactions highlighted a mechanism that could justify the wrong interaction and, for the first time, excluded abnormal mRNA synthesis as a cause for pathology. Instead, our data suggest that the α-thal phenotype could be caused by difficulty in interacting with AHSP causing a degradation of Hb Bernalda. On the contrary, in the case of Hb Southern Italy two different molecular mechanisms were proposed to be involved in the onset of the α-thalassemia phenotype: The reduction of the variant mRNA level by most likely a No-Go Decay and the protein instability likely shifting equilibrium toward AHSP interaction and degradation. The impact on the clinical manifestation due to the presence of the *UGT1A1* mutations in homozygosis in the compound heterozygotes for the Hb Southern Italy is also shown. 

## Figures and Tables

**Table 1:** Hematologic, and biochemical data and α-genotype of the families with the Hb Bernalda/Groene Hart or α1 cod 119 CCT>TCT.

Family n.	Sex/Age (Years)	Relationship	Rbc (10^12^/L)	Hb (g/dL)	Ht (L/L)	MCV (fL)	MCH (pg)	MCHC (%)	S. Iron (µg/dL)	Zpp	Ferritin (ng/mL)	Transferrin (mg/dL)	Bilir Tot (mg/dL)	Bilir Ind (mg/dL)	HbA2 (%)	HbF (%)	α-Globin Genotype
1	F/74	I.2	5.40	15.2	46.7	86.4	28.1	32.5	93	nt	146.0	90	0.59	0.44	nt	nt	αα^Hb Ber^/αα
	F/35	II.2	4.89	13.1	39.8	81.2	26.8	33.1	107	nt	38.0	185	0.43	0.31	3.2	0	αα^Hb Ber^/αα
	M/11	III.1	5.80	13.0	40.5	69.8	22.5	32.2	74	nt	95.0	205	0.46	0.35	3.2	0	αα^Hb Ber^/αα^cod23^
2	M/13	I.1	6.01	14.5	42.8	71.2	24.1	33.9	nt	27	nt	nt	nt	nt	2.0	0	αα^Hb Ber^/αα
3	M/13	I.1	5.53	12.6	38.6	69.8	22.8	32.6	nt	23	nt	nt	nt	nt	3.1	0	αα^Hb Ber^/αα
4	F/46	I.2	4.72	12.8	39.1	82.8	27.1	32.7	75	nt	16.1	240	0.50	0.30	nt	nt	αα^Hb Ber^/αα
	M/13	II.1	5.69	14.9	43.1	75.7	26.2	34.6	123	12	48.7	287	0.50	0.30	3.0	0.2	αα^Hb Ber^/αα
	M/13	II.2	5.94	15.3	45.1	75.9	25.8	33.9	116	12	79.6	256	0.50	0.30	2.9	0	αα^Hb Ber^/αα
5	F/41	I.2	5.54	13.7	42.5	76.7	24.7	32.2	35	nt	8.1	277	0.50	0.30	nt	nt	αα^Hb Ber^/αα
	F/18	II.1	5.10	13.1	38.7	75.9	25.7	33.9	62	nt	26.4	288	0.60	0.40	nt	nt	αα^Hb Ber^/αα
	M/14	II.2	5.57	13.2	41.5	74.5	23.7	31.8	101	26	29.1	311	0.80	0.50	2.5	0	αα^Hb Ber^/αα
6	F/16	I.1	4.27	11.4	34.0	79.6	26.7	33.5	nt	26	nt	nt	nt	nt	2.3	0.3	αα^Hb Ber^/αα
7	M/78	I.1	5.15	15.1	45.4	88.2	29.3	33.3	nt	nt	nt	360	0.82	0.55	2.4	0.5	αα^Hb Ber^/αα
	F/38	II.1	4.85	12.7	36.9	76.1	26.2	34.4	nt	nt	28.0	nt	nt	nt	2.6	0.5	αα^Hb Ber^/αα
8	F/49	I.2	5.10	13.1	39.1	76.7	25.7	33.5	nt	nt	201.0	nt	nt	nt	2.6	0	αα^Hb Ber^/αα
	F/19	II.1	4.90	12.7	37.2	76.0	25.9	34.1	nt	nt	43.0	nt	nt	nt	2.7	0	αα^Hb Ber^/αα
9	M/31	I.1	5.73	15.3	44.3	77.3	26.7	34.5	nt	nt	124.0	nt	nt	nt	2.4	0.5	αα^Hb Ber^/αα
10	M/50	I.1	5.30	14.2	42.0	79.0	27.0	34.0	nt	nt	84.0	nt	nt	nt	2.5	0.2	αα^Hb Ber^/αα
	F/06	II.1	5.66	13.5	40.0	71.0	24.0	34.0	nt	nt	14.0	nt	nt	nt	2.3	0.3	αα^Hb Ber^/αα
11	M/49	I.1	5.83	16.0	47.8	82.0	27.4	33.5	nt	nt	117.0	255	1.17	0.83	2.8	0.5	αα^Hb Ber^/αα
	F/23	II.1	5.16	14.0	41.4	80.2	27.1	33.8	nt	nt	45.0	296	0.49	0.34	2.7	0	αα^Hb Ber^/αα
12	M/37	I.1	5.43	14.2	43.4	79.9	26.2	32.7	nt	nt	92.0	nt	nt	nt	3.1	0.5	αα^Hb Ber^/αα
13	F/43	I.2	5.25	11.6	35.9	68.4	22.1	32.3	nt	nt	4.00	nt	nt	nt	2.6	0.5	αα^Hb Ber^/αα
	M/18	II.1	6.01	15.8	46.7	77.7	26.3	33.8	nt	nt	27.0	nt	nt	nt	2.9	0.5	αα^Hb Ber^/αα
14	F/57	I.2	5.60	14.1	42.6	76.1	25.2	33.1	nt	nt	48.0	nt	nt	nt	2.5	0.5	αα^Hb Ber^/αα
	F/37	II.1	4.88	12.5	37.6	77.0	25.6	33.2	nt	nt	17.0	nt	nt	nt	2.3	0.5	αα^Hb Ber^/αα
	F/30	II.2	4.18	11.1	33.3	79.7	26.6	33.3	nt	nt	24.0	nt	nt	nt	2.7	0.5	αα^Hb Ber^/αα
15	M/32	I.1	5.51	14.5	43.1	78.2	26.3	33.6	nt	nt	82.0	nt	nt	nt	3.0	0.5	αα^Hb Ber^/αα
16	M/43	I.1	5.55	14.8	43.4	78.2	26.7	34.1	nt	nt	159.0	nt	nt	nt	2.4	0.8	αα^Hb Ber^/αα
17	M/42	I.1	5.90	15.8	46.9	79.5	26.8	33.7	nt	nt	95.0	nt	nt	nt	3.1	0.5	αα^Hb Ber^/αα

Family n.: Family number; Rbc: Red Blood Cells; Hb: Hemoglobin; Ht: Hematocrit; MCV: Mean Corpuscular Volume; MCH: Mean Corpuscular Hemoglobin; MCHC: Mean Corpuscular Hemoglobin Concentration; S. iron: Serum iron; Zpp: Zinc Protoporphyrin; Bilir tot: Total bilirubin; Bilir ind: Indirect Bilirubin; nt: not tested; αα^Hb Ber^: Hb Bernalda/Groene Hart; αα^cod23^: α1 cod 23 GAT>TAG.

**Table 2:** Hematologic, and biochemical data and α-genotype of the families with the Hb Southern Italy (Hb Caserta, α2 cod 26 GCG>ACG, in *cis* to the Hb Sun Prairie, α2 cod 130 GCT>CCT).

Family n.	Sex/Age (Years)	Relationship	Rbc (10^12^/L)	Hb (g/dL)	Ht (L/L)	MCV (fL)	MCH (pg)	MCHC (%)	Ret (%)	Erythro Morpho	S. Iron (µg/dL)	Zpp	Ferritin (ng/mL)	Transf (mg/dL)	Bilir Tot (mg/dL)	Bilir Ind (mg/dL)	HbA2 (%)	Hb F (%)	α-Globin Genotype
18	M/adult	I.1	5.85	14.6	45.3	77.4	25.0	32.3	3.40	nt	139	nt	169.0	nt	nt	nt	2.1	0	αα^Hb SI^/αα *
19	M/61	I.1	5.61	14.6	44.1	78.6	26.0	33.1	nt	nt	135	nt	100.0	nt	0.92	0.75	2.1	0.3	αα^Hb SI^/αα
	F/59	I.2	5.03	13.6	40.0	79.5	27.0	34.0	nt	nt	90	nt	44.0	nt	1.70	nt	2.4	0.3	−α^3.7^/αα
	M/22 ^§^	II.1	6.17	12.8	41.1	66.7	20.7	31.0	nt	nt	81	nt	113.0	nt	4.70	nt	2.2	0.3	αα^Hb SI^/−α^3.7^
	M/32 ^§^	II.1	6.06	12.9	39.5	65.2	21.3	32.7	nt	nt	95	nt	178.0	nt	5.30	4.86	2.0	0.2	
	M/30	II.2	5.32	13.3	39.3	73.9	25.0	33.8	nt	nt	25	nt	185.0	nt	1.13	0.92	2.5	0.2	αα^Hb SI^/αα
20	M/adult	I.1	6.69	16.5	47.9	71.7	24.7	34.4	2.23	nt	84	nt	191.0	nt	0.70	nt	2.2	0.2	αα^Hb SI^/αα
	M/	II.1	5.37	10.0	32.4	60.3	18.5	30.7	nt	nt	35	nt	26.0	nt	0.50	nt	2.1	0.2	αα^Hb SI^/αα
21	M/13	I.1	5.45	13.5	40.8	74.9	24.8	33.1	nt	nt	nt	15	nt	nt	nt	nt	2.9	0.6	αα^Hb SI^/αα
22	F/18	I.1	5.01	11.8	35.7	71.0	23.6	33.1	nt	nt	66	nt	51.0	224	nt	nt	2.3	0	αα^Hb SI^/αα
23	M/65	I.1	5.98	14.7	44.0	74.0	24.0	33.0	nt	A	nt	nt	348.0	nt	nt	nt	2.6	0.2	αα^Hb SI^/αα
	F/55	I.2	5.40	13.1	40.0	73.0	24.0	33.0	nt	nt	nt	nt	72.0	nt	nt	nt	2.3	0	αα^IVSI-5nt^/αα
	M/34	II.1	5.29	10.5	34.0	64.0	20.0	31.0	nt	A	nt	nt	225.0	nt	nt	nt	2.1	0.5	αα^Hb SI^/αα^IVSI-5nt^
24	F/38	I.1	4.73	12.2	36.0	76.2	25.8	33.9	nt	nt	nt	nt	127.0	nt	nt	nt	2.9	0	αα^Hb SI^/αα
25	F/26	I.1	5.07	12.7	37.1	73.0	25.0	34.2	nt	nt	nt	nt	21.0	nt	nt	nt	2.6	0.6	αα^Hb SI^/αα
26	F/54	I.2	5.15	13.3	38.9	75.0	25.8	34.3	nt	nt	91	nt	nt	nt	1.44	nt	2.5	0	αα^Hb SI^/αα
	M/24	II.1	5.94	14.9	43.6	73.0	25.1	34.3	nt	nt	51	nt	nt	nt	0.54	nt	2.3	0	αα^Hb SI^/αα
27	F/59	I.2	5.56	13.5	41.0	74.0	24.3	32.9	0.90	nt	96	nt	95.6	229	0.62	nt	2.4	0	αα^Hb SI^/αα
	M/34	II.1	5.52	13.7	41.7	75.0	24.8	32.8	nt	nt	99	nt	160.8	nt	0.94	nt	2.7	0	αα^Hb SI^/αα
	F/26	II.2	5.18	12.9	40.2	77.0	24.9	32.2	1.20	nt	134	nt	36.0	241	1.17	nt	2.6	0.6	αα^Hb SI^/αα

Family n.: Family number; Rbc: Red Blood Cells; Hb: Hemoglobin; Ht: Hematocrit; MCV: Mean Corpuscular Volume; MCH: Mean Corpuscular Hemoglobin; MCHC: Mean Corpuscular Hemoglobin Concentration; Ret: Reticulocytes; Erythro morpho: Erythrocytes morphology; S. iron: Serum Iron; Zpp: Zinc Protoporphyrin; Transf: Transferrin; Bilir tot: Total Bilirubin; Bilir ind: Indirect Bilirubin; * non-α/α biosynthetic ratio = 0.64; § same person; αα^Hb SI^: α2 Hb Southern Italy; A: Anisocytosis; nt: Not tested.

**Table 3:** Stability analysis of the tetrameric human deoxy hemoglobin (PDB code 2HHB) and of the Hb Southern Italy model.

Protein	Energy (kcal/mol)	Protein	Energy (kcal/mol)
Wild-type tetramer	−161	α^Hb SI^βα^Hb SI^β	−156.5
A (α-globin chain)	−21.34	A (α-globin chain Hb SI)	−17.12
B (β-globin chain)	−27.04	B (β-globin chain)	−27.04
C (α-globin chain)	−17.71	C (α-globin chain Hb SI)	−15.05
D (β-globin chain)	−21.19	D (β-globin chain)	−21.19

**Table 4:** Frequencies of Hb Bernalda/Groene Hart and Hb Southern Italy in Italian regions or provinces. The number of alleles are reported in parentheses.

Region	Napoli	Matera	Sicily/Agrigento	Southern Italy
Hb Bernalda/Groene Hart				
Relative α-thal frequencies	0.5% (1)	2.0% (5)	1.8% (11)/4.6% (9)	1.6% (17)
Non-deletional α-thal frequencies	3.0%	7.9%	5.8%/16.1%	
Hb Southern Italy				
Relative α-thal frequencies	3.2% (6)	0.4% (1)	0.5% (3)	0.9% (10)
Non-deletional α-thal frequencies	20.0%	1.6%	1.6%	

## Supplementary Materials

The following are available online at https://www.mdpi.com/2073-4425/11/8/870/s1. Figure S1: Position of mutations for Hb Bernalda/Groene Hart and Hb Southern Italy variants; Figure S2: Hydrogen bonds between AHSP and α-globin chain highlighted with the Swiss PDB viewer program; Figure S3: Secondary structure of α2 Southern Italy mRNA relative to the normal α2 mRNA; Table S1: Oligonucleotides used as Primers in the reported applications; Table S2: Type of amino acids present in the α-globin chain; Table S3: Type of amino acids present in the β-globin chain.
