# Supplementary material for: Effect of Mutations on mRNA and Globin Stability: The Cases of Hb Bernalda/Groene Hart and Hb Southern Italy
Source: Genes (Basel). 2020 Jul 31;11(8):870. doi: 10.3390/genes11080870 (PMC7466077; doi:10.3390/genes11080870)
Supplement: Supplementary file 1 [file genes-11-00870-s001.pdf]

Supplementary material

Effect of Mutations on mRNA and Globin Stability: The Cases of Hb Bernalda/Groene Hart and Hb Southern Italy

Giovanna Cardiero [1], Gennaro Musollino [1], Maria Grazia Friscia [2], Rosario Testa [3], Lucrezia Virruso [4], Caterina Di Girgenti [4], Mercedes Caldora [5], Rosario Colella Bisogno [6], Carlo Gaudiano [7], Giuseppe Manco [8] and Giuseppina Lacerra [1,*]

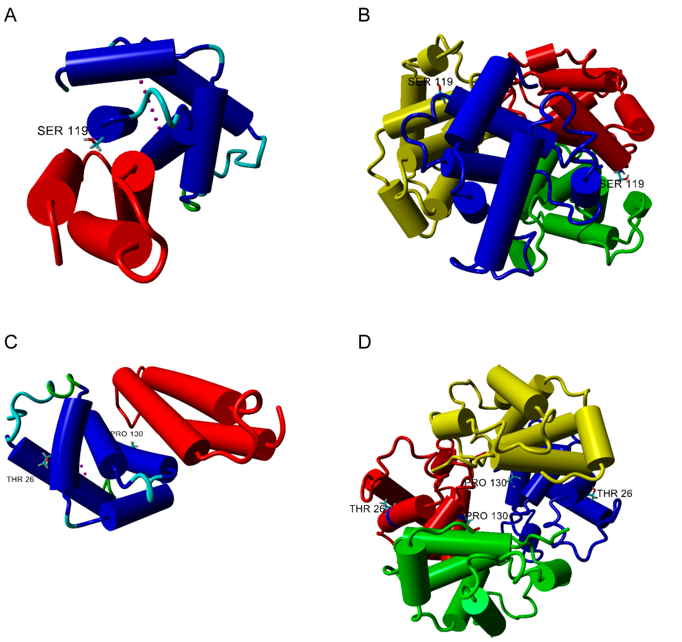

Figure S1. Position of mutations for Hb Bernalda/Groene Hart and Hb Southern Italy variants. (**A**) and (**B**) Positions of Ser119 in 1Y01 and 2HHB, respectively. (**C**) and (**D**) Positions of Thr26 and Pro130 in 1Y01 and 2HHB, respectively. The chains were in different colors. Highlighted residues are in stick representation. Pictures were obtained by the Yasara program.

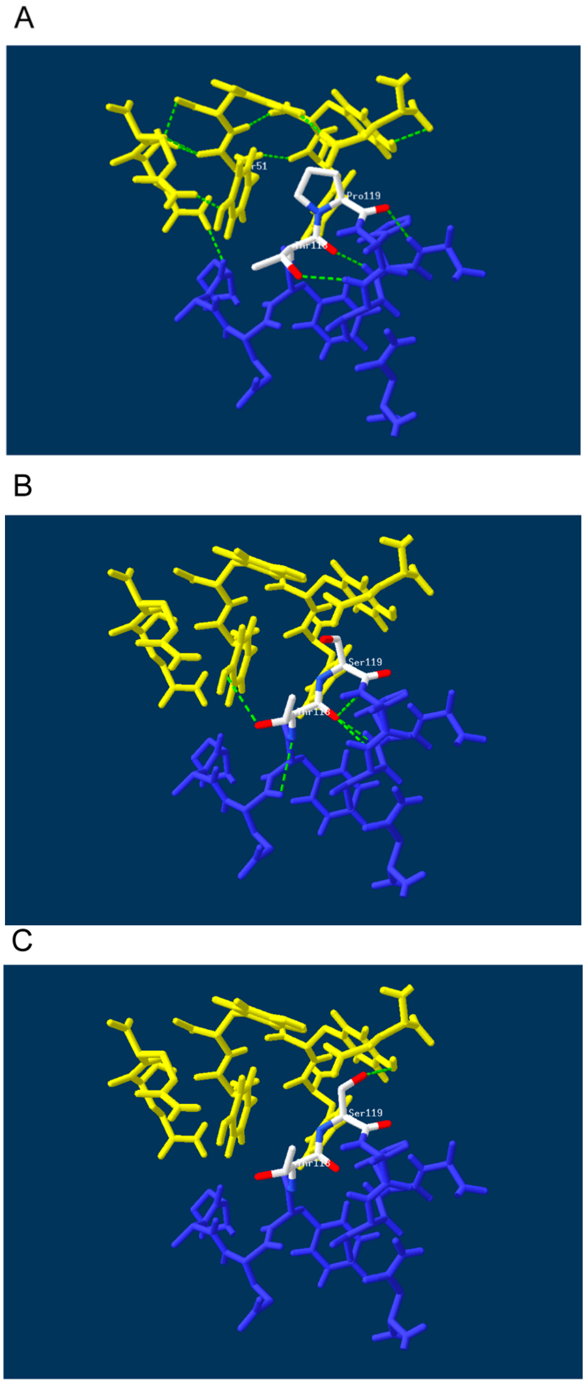

Figure S2. Hydrogen bonds between AHSP and α-globin chain highlighted with the Swiss PDB viewer program. (**A**) The chains interface in 1Y01 showing hydrogen bond interactions. (**B**) The mutant model highlighted with the hydrogen bond of Thr118 with Tyr51. (**C**) Hydrogen bond formed by the Ser119 rotamer with Tyr48.

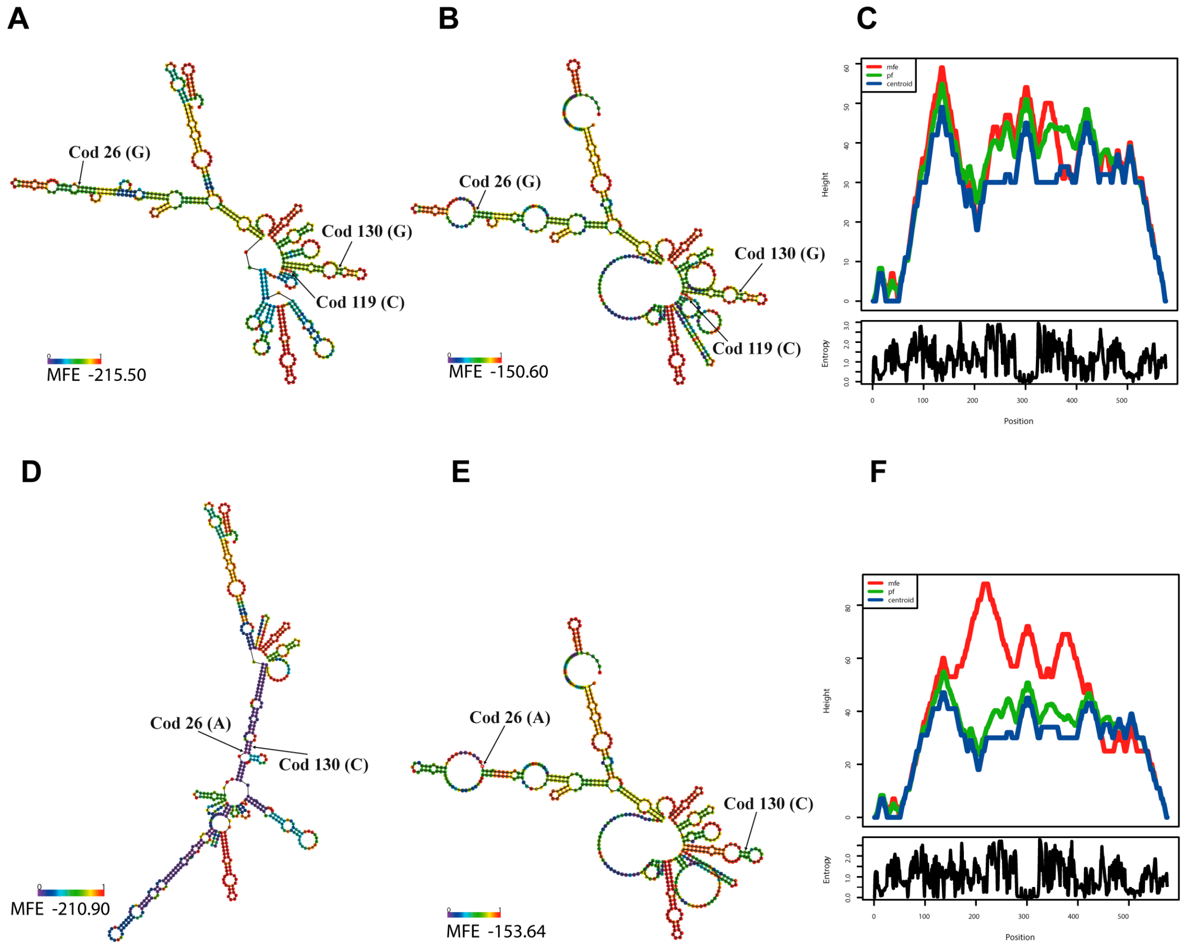

MFE secondary structure

Centroid secondary structure

MFE=minimum free energy

Figure S3. Secondary structure of α2-globin mRNAs predicted by means of the RNAfold web server http://rna.tbi.univie.ac.at/cgi-bin/RNAWebSuite/RNAfold.cgi [26] (**A**,**B**,**C**), normal α2-globin mRNA; (**D**,**E**,**F**) α2 Hb Southern Italy mRNA. For each α-globin mRNA has been reported the minimum free energy (MFE) secondary structure (**A**,**D**), the Centroid secondary structure (**B**,**E**), with the corresponding values, and the mountain plot representation (**C**,**F**) of the MFE structure, the thermodynamic ensemble of RNA structures, and the centroid structure.

Table S1. Oligonucleotides used as Primers in the reported applications. The sequence and position of the primers were from GenBank Sequence *NG_000006.1.

Name	Direction, Sequence	Position from the Cap Site	Application	Used with Primer	Amplicon Length (bp)	References
A For	5′-GCCCTGAGCGACCTGCACGCG-3′	α1, α2 +401/+421	DGGE DNA	B	α1 301; α2 294	[20]
B Rev	5′-ACAGAAGCCAGGAACTTGTC-3′	α1 +682/+701; α2 +675/+694	DGGE DNA			[20]
C For	5′-TGGAGGGTGGAGACGTCCTG-3′	α1, α2 −202/−183	DGGE DNA, PCR	D, F, G	α1, α2 441; α1 975; α2 1106	[20]
D Rev	5′-AAGCAGAGTGAGGGGTGGGG-3′	α1, α2 +220/+239	DGGE DNA			[20]
E For	5′-TCGCGGCCCGGCACTCTTCTG-3′	α1, α2 −12/+9	Seq α1, α2			[22]
F Rev	5′-GAGGCCCAAGGGGCAAGAAGCAT-3′	α1 +751/+773	α1 Seq, RT-PCR			[13]
G Rev	5′-GTCTGAGACAGGTAAACACCTCCAT-3′	α2 +880/+904	Seq α2, Common ARMS			[19]
H For	5′-CACAGACTCAGAGAGAACC-3′	α1, α2 +15/+33	RT-PCR α1, α2	F, I	α1 493; α2 261	[21]
I Rev	5′-CGTTGGGCATGTCGTCCAC-3′	α2 +374/+392	RT-PCR α2			[22]
J For	5′-CCTCCCCGCCGAGTTCACCC-3′	α2 +635/+654	RE cDNA analysis	K	132 bp	p.a.
K Rev	5′-GGGAGGCCCATCGGGCAGGAGGAAC-3′	α2 +744/+768	RT-PCR α2<br>RE cDNA analysis			[34]
L For	5′-GGCAAGAAGGTGGCCGAC-3′	α1 +332/+349	DGGE cDNA α1	B	α1 221	[21]
M For, Hb Cas	5′GCACGCTGGCGAGTATG**C**T**A**-3′	α1, α2 +97/+116	ARMS	G	808	[13]
N For, cod26nor	5′-GCACGCTGGCGAGTATG**C**TG-3′	α1, α2 +97/+116	ARMS	G	808	[13]
O For, Hb Ber	5′-CCTCCCCGCCGAGTTCA**G**C**T**-3′	α1, α2 +642/+661	ARMS	R	α1 275	p.a.
P For, Hb S.P.	5′-CTCCCTGGACAAGTTC**G**TG**C**-3′	α1, α2 +668/+687	ARMS	G	237	[13]
Q For cod130nor	5′-CTCCCTGGACAAGTTC**G**TGG-3′	α1, α2 +668/+687	ARMS	G	237	[13]
R Rev	5′-TGTGTGTCCCAGCTGCTGTCCACGC-3′	α1 +892/+916	Common ARMS			[13]
S For	5′-AGGCTGTGGGCAGAGTCAGAAGA-3′	35943-35965 *	Control ARMS	T	714	[13]
T Rev	5′-CAATAGCTGGAACCGGCTGGAG-3′	36656-36635 *	Control ARMS			[13]
U For	5′-CCCAGAGCCAGGTTTGTTTATCTG-3′	32840-32863 *	RFLP RsaI	G	1803	[21]
UGT1A1 For	5′-AAGTGAACTCCCTGCTACCTT-3′	UGT1A1 −130/−110	PCR, Seq	UGT1A1 Rev	253	[24]
UGT1A1 Rev	5′-CCACTGGGATCAACAGTATCT-3′	UGT1A1 +104/+124	PCR, Seq			[24]

Rev: Reverse; For: Forward; Seq: sequencing analysis; Hb Cas: Hb Caserta; Hb Ber: Hb Bernalda; Hb S.P.: Hb Sun Prairie; RE cDNA analysis: Restriction enzyme cDNA analysis; p.a.: present article.

Table S2. (**A**) Nucleotide triplets of the coding α-globin mRNA (NM_000517.6), starting from the ATG until the stop codon, below the relative amino acid present in the α-globin chain (NP_000508.1). (**B**) Type of amino acids present in the α-globin chain, the corresponding coding triplet, and number of times present in the α-globin mRNA.

A

ATG	GTG	CTG	TCT	CCT	GCC	GAC	AAG	ACC	AAC	GTC	AAG	GCC	GCC	TGG	GGT	AAG	GTC	GGC	GCG
M	V	L	S	P	A	D	K	T	N	V	K	A	A	W	G	K	V	G	A
CAC	GCT	GGC	GAG	TAT	GGT	GCG	GAG	GCC	CTG	GAG	AGG	ATG	TTC	CTG	TCC	TTC	CCC	ACC	ACC
H	A	G	E	Y	G	A	E	A	L	E	R	M	F	L	S	F	P	T	T
AAG	ACC	TAC	TTC	CCG	CAC	TTC	GAC	CTG	AGC	CAC	GGC	TCT	GCC	CAG	GTT	AAG	GGC	CAC	GGC
K	T	Y	F	P	H	F	D	L	S	H	G	S	A	Q	V	K	G	H	G
AAG	AAG	GTG	GCC	GAC	GCG	CTG	ACC	AAC	GCC	GTG	GCG	CAC	GTG	GAC	GAC	ATG	CCC	AAC	GCG
K	K	V	A	D	A	L	T	N	A	V	A	H	V	D	D	M	P	N	A
CTG	TCC	GCC	CTG	AGC	GAC	CTG	CAC	GCG	CAC	AAG	CTT	CGG	GTG	GAC	CCG	GTC	AAC	TTC	AAG
L	S	A	L	S	D	L	H	A	H	K	L	R	V	D	P	V	N	F	K
CTC	CTA	AGC	CAC	TGC	CTG	CTG	GTG	ACC	CTG	GCC	GCC	CAC	CTC	CCC	GCC	GAG	TTC	ACC	CCT
L	L	S	H	C	L	L	V	T	L	A	A	H	L	P	A	E	F	T	P
GCG	GTG	CAC	GCC	TCC	CTG	GAC	AAG	TTC	CTG	GCT	TCT	GTG	AGC	ACC	GTG	CTG	ACC	TCC	AAA
A	V	H	A	S	L	D	K	F	L	A	S	V	S	T	V	L	T	S	K
TAC	CGT	TAA																	
	R	Stop																	

B

**AA**	**n.**	**Codon (Time Present)**
Ala, A	21	GCC(12), GCG(7), GCT(2), GCA(0)
Arg, R	3	CGT(1), CGG(1), AGG(1), CGC(0), CGA(0), AGA(0)
Asn, N	4	AAC(4), AAT(0)
Asp, D	8	GAC(8), GAT(0)
Cys, C	1	TGC(1), TGT(0)
Gln, Q	1	CAG(1), CAA(0)
Glu, E	4	GAG(4), GAA(0)
Gly, G	7	GGC(5), GGT(2), GGA(0), GGG(0)
His, H	10	CAC(10), CAT(0)
Ile, I	0	ATT(0), ATC(0), ATA(0)
Start	1	ATG(1), GTG(0)
Leu, L	18	CTG (14), CTC (2), CTT(1), CTA(1), TTA(0), TTG(0)
Lys, K	11	AAG(10), AAA(1)
Met, M	2	ATG (2)
Phe, F	7	TTC(7), TTT(0)
Pro, P	7	CCC(3), CCT(2), CCG(2), CCA(0)
Ser, S	11	TCC(4), AGC(4), TCT(3), TCA(0), TCG(0), AGT(0)
Thr, T	9	ACC(9), ACT(0), ACA(0), ACG(0)
Trp, W	1	TGG(1)
Tyr, Y	3	TAC(2), TAT(1)
Val, V	13	GTG(9), GTC(3), GTT(1), GTA(0)
Stop	1	TAG(0), TGA(0), TAA(1)

Table S3. (**A**) Nucleotide triplets of the coding α-globin mRNA (NM_000518.5), starting from the ATG until the stop codon, below the relative amino acid present in the α-globin chain (NP_000509.1). (**B**) Type of amino acids present in the α-globin chain, the corresponding coding triplet, and number of times present in the α-globin mRNA

A

ATG	GTG	CAT	CTG	ACT	CCT	GAG	GAG	AAG	TCT	GCC	GTT	ACT	GCC	CTG	TGG	GGC	AAG	GTG	AAC
M	V	H	L	T	P	E	E	K	S	A	V	T	A	L	W	G	K	V	N
GTG	GAT	GAA	GTT	GGT	GGT	GAG	GCC	CTG	GGC	AGG	CTG	CTG	GTG	GTC	TAC	CCT	TGG	ACC	CAG
V	D	E	V	G	G	E	A	L	G	R	L	L	V	V	Y	P	W	T	Q
AGG	TTC	TTT	GAG	TCC	TTT	GGG	GAT	CTG	TCC	ACT	CCT	GAT	GCT	GTT	ATG	GGC	AAC	CCT	AAG
R	F	F	E	S	F	G	D	L	S	T	P	D	A	V	M	G	N	P	K
GTG	AAG	GCT	CAT	GGC	AAG	AAA	GTG	CTC	GGT	GCC	TTT	AGT	GAT	GGC	CTG	GCT	CAC	CTG	GAC
V	K	A	H	G	K	K	V	L	G	A	F	S	D	G	L	A	H	L	D
AAC	CTC	AAG	GGC	ACC	TTT	GCC	ACA	CTG	AGT	GAG	CTG	CAC	TGT	GAC	AAG	CTG	CAC	GTG	GAT
N	L	K	G	T	F	A	T	L	S	E	L	H	C	D	K	L	H	V	D
CCT	GAG	AAC	TTC	AGG	CTC	CTG	GGC	AAC	GTG	CTG	GTC	TGT	GTG	CTG	GCC	CAT	CAC	TTT	GGC
P	E	N	F	R	L	L	G	N	V	L	V	C	V	L	A	H	H	F	G
AAA	GAA	TTC	ACC	CCA	CCA	GTG	CAG	GCT	GCC	TAT	CAG	AAA	GTG	GTG	GCT	GGT	GTG	GCT	AAT
K	E	F	T	P	P	V	Q	A	A	Y	Q	K	V	V	A	G	V	A	N
GCC	CTG	GCC	CAC	AAG	TAT	CAC	TAA												
	L	A	H	K	Y	H	Stop												

B

AA	n	Codon (n of Times Present)
Ala, A	15	GCC(9),GCT(6), GCA(0), GCG(0)
Arg, R	3	AGG(3), CGT(0), CGC(0), CGA(0), CGG(0), AGA(0)
Asn, N	6	AAC(5). AAT(1)
Asp, D	7	GAT(5), GAC(2)
Cys, C	2	TGT(2), TGC(0)
Gln, Q	3	CAG(3), CAA(0)
Glu, E	8	GAG(6), GAA(2)
Gly, G	13	GGC(8), GGT(4), GGG(1), GGA(0)
His, H	9	CAC(6), CAT(3)
Ile, I	0	ATT(0), ATC(0), ATA(0)
Start	1	ATG(1), GTG(0)
Leu, L	18	CTG(15), CTC(3), TTA(0), TTG(0), CTT(0), CTA(0)
Lys, K	11	AAG(8), AAA(3)
Met, M	1	ATG (1)
Phe, F	8	TTT(5), TTC(3)
Pro, P	7	CCT(5), CCA(2), CCC(0), CCG(0)
Ser, S	5	AGT(2), TCC(2), TCT(1), TCA(0), TCG(0), AGC(0)
Thr, T	7	ACT(3), ACC(3), ACA(1), ACG(0)
Trp, W	2	TGG(2)
Tyr, Y	3	TAT(2), TAC(1)
Val, V	18	GTG(13), GTT(3), GTC(2), GTA(0)
Stop	1	TAA(1), TAG(0), TGA(0)
